# 4-Hydr­oxy-*N*′-(2-hydr­oxy-3-methoxy­benzyl­idene)benzohydrazide mono­hydrate

**DOI:** 10.1107/S1600536808030894

**Published:** 2008-09-27

**Authors:** Jiu-Fu Lu

**Affiliations:** a School of Chemistry and Environmental Science, Shaanxi University of Technology, Hanzhong 723000, People’s Republic of China

## Abstract

In the title compound, C_15_H_14_N_2_O_4_·H_2_O, the dihedral angle between the two aromatic rings is 33.3 (5)°. The meth­oxy group is twisted slightly away from the attached benzene ring [C—O—C—C = 13.8 (9)°]. An intra­molecular O—H⋯N hydrogen bond is observed. In the crystal structure, the mol­ecules are linked into a two-dimensional network parallel to the (010) plane by inter­molecular N—H⋯O, O—H⋯O and C—H⋯O hydrogen bonds

## Related literature

For related structures, see: Lu *et al.* (2008*a* ▶,*b* ▶,*c* ▶); Nie (2008 ▶); He (2008 ▶); Shi *et al.* (2007 ▶). For bond-length data, see: Allen *et al.* (1987 ▶).
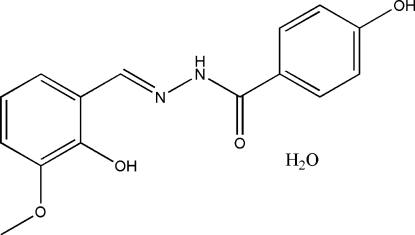


## Experimental

### 

#### Crystal data

C_15_H_14_N_2_O_4_·H_2_O
*M* *_r_* = 304.30
Monoclinic, 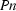

*a* = 4.891 (2) Å
*b* = 12.171 (5) Å
*c* = 12.371 (5) Å
β = 95.724 (7)°
*V* = 732.8 (5) Å^3^
*Z* = 2
Mo *K*α radiation
μ = 0.10 mm^−1^
*T* = 298 (2) K
0.08 × 0.07 × 0.07 mm

#### Data collection

Bruker APEXII CCD area-detector diffractometer
Absorption correction: multi-scan (*SADABS*; Sheldrick, 2004 ▶) *T*_min_ = 0.992, *T*_max_ = 0.993
5972 measured reflections
1582 independent reflections
791 reflections with *I* > 2σ(*I*)
*R*_int_ = 0.086

#### Refinement

*R*[*F* ^2^ > 2σ(*F* ^2^)] = 0.051
*wR*(*F* ^2^) = 0.119
*S* = 0.92
1582 reflections
211 parameters
6 restraints
H atoms treated by a mixture of independent and constrained refinement
Δρ_max_ = 0.14 e Å^−3^
Δρ_min_ = −0.15 e Å^−3^

### 

Data collection: *APEX2* (Bruker, 2004 ▶); cell refinement: *SAINT* (Bruker, 2004 ▶); data reduction: *SAINT*; program(s) used to solve structure: *SHELXS97* (Sheldrick, 2008 ▶); program(s) used to refine structure: *SHELXL97* (Sheldrick, 2008 ▶); molecular graphics: *SHELXTL* (Sheldrick, 2008 ▶); software used to prepare material for publication: *SHELXTL*.

## Supplementary Material

Crystal structure: contains datablocks global, I. DOI: 10.1107/S1600536808030894/ci2678sup1.cif

Structure factors: contains datablocks I. DOI: 10.1107/S1600536808030894/ci2678Isup2.hkl

Additional supplementary materials:  crystallographic information; 3D view; checkCIF report

## Figures and Tables

**Table 1:** Hydrogen-bond geometry (Å, °)

*D*—H⋯*A*	*D*—H	H⋯*A*	*D*⋯*A*	*D*—H⋯*A*
O1—H1⋯N1	0.82	1.99	2.687 (5)	142
N2—H2⋯O5^i^	0.90 (3)	1.99 (3)	2.820 (5)	154 (5)
O4—H4⋯O2^ii^	0.82	2.15	2.872 (7)	147
O5—H5*A*⋯O3^iii^	0.86 (3)	1.97 (3)	2.769 (6)	156 (6)
O5—H5*B*⋯O3^iv^	0.84 (4)	2.01 (3)	2.769 (6)	148 (5)
C7—H7⋯O5^i^	0.93	2.48	3.229 (7)	138
C14—H14⋯O5^i^	0.93	2.34	3.218 (7)	158

Symmetry codes: (i) 
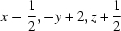
; (ii) 
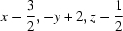
; (iii) 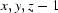; (iv) 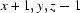.

**Fig. 1.**
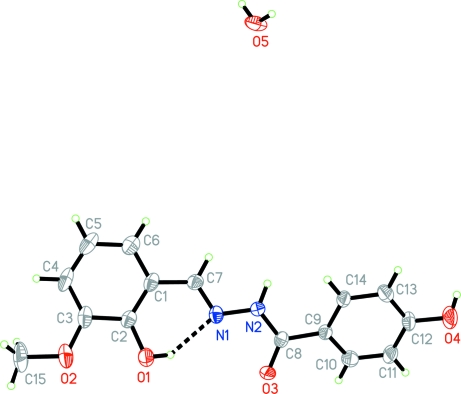
The molecular structure of the title compound, showing 30% probability displacement ellipsoids and the atom-numbering scheme. The dashed line indicates a hydrogen bond.

**Fig. 2.**
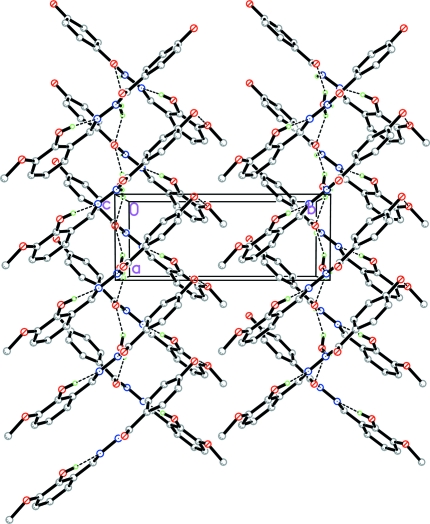
The crystal packing of the title compound, viewed along the c axis. H atoms not involved in hydrogen bonding (dashed lines) have been omitted for clarity.

## supplementary crystallographic information

### Comment

As part of our investigation of the crystal structures of Schiff bases derived from the condensation of aldehydes with benzohydrazides (Lu *et al.*, 2008*a*,b,c), we report here the crystal structure of the title new Schiff base compound.

The asymmetric unit of the title compound (Fig. 1), consists of a Schiff base molecule and a water molecule of crystallization. The bond lengths have normal values (Allen *et al.*,  1987), and are comparable to those observed in similar compounds (Nie, 2008; He, 2008; Shi *et al.*, 2007). The dihedral angle between the two aromatic rings is 33.3 (5)°, indicating that the Schiff base molecule is twisted. An intramolecular O—H···N hydrogen bond is observed.

In the crystal structure, the molecules are linked into a two-dimensional network parallel to the (010) by intermolecular N—H···O, O—H···O and C—H···O hydrogen bonds (Table 1).

### Experimental

The title compound was prepared by the Schiff base condensation of 2-hydroxy-3-methoxybenzaldehyde (0.1 mol) and 4-hydroxybenzohydrazide (0.1 mmol) in 95% ethanol (50 ml). The excess ethanol was removed by distillation. The colourless solid obtained was filtered and washed with ethanol. Single crystals suitable for X-ray diffraction were obatined by slow evaporation of a 95% ethanol solution at room temperature.

### Refinement

The imino and water H atoms were located in a difference map and refined with N-H, O-H and H···H distances restrained to 0.90 (1), 0.85 (1), and 1.37 (2) Å, respectively. The other H atoms were positioned geometrically (C-H = 0.93-0.96 Å and O-H = 0.82 Å) and refined using a riding model, with U_iso_(H) = 1.2U_eq_(C) and 1.5U_eq_(C_methyl_ and O). A rotating group model was used for methyl and hydroxyl groups. In the absence of significant anomalous scattering effects, Friedel pairs were averaged.

### Crystal data

**Table:**

C_15_H_14_N_2_O_4_·H_2_O	*F*(000) = 320
*M**_r_* = 304.30	*D*_x_ = 1.379 Mg m^−^^3^
Monoclinic, *P**n*	Mo *K*α radiation, λ = 0.71073 Å
Hall symbol: P -2yac	Cell parameters from 623 reflections
*a* = 4.891 (2) Å	θ = 2.3–24.0°
*b* = 12.171 (5) Å	µ = 0.11 mm^−^^1^
*c* = 12.371 (5) Å	*T* = 298 K
β = 95.724 (7)°	Block, colourless
*V* = 732.8 (5) Å^3^	0.08 × 0.07 × 0.07 mm
*Z* = 2	

### Data collection

**Table:**

Bruker APEXII CCD area-detector diffractometer	1582 independent reflections
Radiation source: fine-focus sealed tube	791 reflections with *I* > 2σ(*I*)
graphite	*R*_int_ = 0.086
ω scans	θ_max_ = 27.0°, θ_min_ = 1.7°
Absorption correction: multi-scan (SADABS; Sheldrick, 2004)	*h* = −6→6
*T*_min_ = 0.992, *T*_max_ = 0.993	*k* = −15→15
5972 measured reflections	*l* = −15→15

### Refinement

**Table:**

Refinement on *F*^2^	Primary atom site location: structure-invariant direct methods
Least-squares matrix: full	Secondary atom site location: difference Fourier map
*R*[*F*^2^ > 2σ(*F*^2^)] = 0.051	Hydrogen site location: inferred from neighbouring sites
*wR*(*F*^2^) = 0.119	H atoms treated by a mixture of independent and constrained refinement
*S* = 0.92	*w* = 1/[σ^2^(*F*_o_^2^) + (0.0473*P*)^2^] where *P* = (*F*_o_^2^ + 2*F*_c_^2^)/3
1582 reflections	(Δ/σ)_max_ = 0.001
211 parameters	Δρ_max_ = 0.14 e Å^−^^3^
6 restraints	Δρ_min_ = −0.15 e Å^−^^3^

### Special details

**Table:**

Geometry. All esds (except the esd in the dihedral angle between two l.s. planes) are estimated using the full covariance matrix. The cell esds are taken into account individually in the estimation of esds in distances, angles and torsion angles; correlations between esds in cell parameters are only used when they are defined by crystal symmetry. An approximate (isotropic) treatment of cell esds is used for estimating esds involving l.s. planes.
Refinement. Refinement of F^2^ against ALL reflections. The weighted R-factor wR and goodness of fit S are based on F^2^, conventional R-factors R are based on F, with F set to zero for negative F^2^. The threshold expression of F^2^ > 2sigma(F^2^) is used only for calculating R-factors(gt) etc. and is not relevant to the choice of reflections for refinement. R-factors based on F^2^ are statistically about twice as large as those based on F, and R- factors based on ALL data will be even larger.

### Fractional atomic coordinates and isotropic or equivalent isotropic displacement parameters (Å^2^)

**Table:**

	*x*	*y*	*z*	*U*_iso_*/*U*_eq_	
O1	0.2886 (8)	0.7446 (3)	0.9901 (3)	0.0588 (11)	
H1	0.2167	0.8048	0.9782	0.088*	
O2	0.6029 (9)	0.5752 (3)	1.0195 (4)	0.0747 (13)	
O3	−0.1590 (7)	1.0380 (3)	0.9939 (3)	0.0564 (10)	
O4	−0.9973 (10)	1.3626 (4)	0.7359 (3)	0.0823 (15)	
H4	−1.0197	1.3619	0.6694	0.123*	
O5	0.3665 (9)	1.0067 (4)	0.0948 (3)	0.0717 (13)	
N1	0.1007 (8)	0.9097 (3)	0.8590 (3)	0.0460 (11)	
N2	−0.0723 (9)	0.9954 (4)	0.8239 (3)	0.0454 (11)	
C1	0.4174 (11)	0.7745 (4)	0.8075 (5)	0.0494 (14)	
C2	0.4327 (11)	0.7168 (4)	0.9064 (5)	0.0463 (14)	
C3	0.6063 (12)	0.6245 (5)	0.9194 (5)	0.0579 (17)	
C4	0.7584 (13)	0.5927 (5)	0.8385 (6)	0.072 (2)	
H4A	0.8732	0.5319	0.8484	0.087*	
C5	0.7435 (13)	0.6500 (6)	0.7418 (6)	0.073 (2)	
H5	0.8484	0.6279	0.6870	0.088*	
C6	0.5746 (12)	0.7393 (5)	0.7267 (5)	0.0594 (16)	
H6	0.5647	0.7771	0.6611	0.071*	
C7	0.2392 (11)	0.8680 (4)	0.7868 (4)	0.0485 (14)	
H7	0.2243	0.8993	0.7180	0.058*	
C8	−0.2022 (10)	1.0526 (4)	0.8945 (5)	0.0421 (13)	
C9	−0.4033 (10)	1.1358 (4)	0.8497 (4)	0.0410 (13)	
C10	−0.5053 (12)	1.2129 (5)	0.9167 (5)	0.0589 (16)	
H10	−0.4389	1.2138	0.9898	0.071*	
C11	−0.6996 (13)	1.2877 (5)	0.8800 (5)	0.0694 (19)	
H11	−0.7650	1.3381	0.9277	0.083*	
C12	−0.7996 (11)	1.2885 (5)	0.7710 (5)	0.0558 (16)	
C13	−0.7035 (13)	1.2128 (5)	0.7021 (5)	0.0655 (18)	
H13	−0.7698	1.2125	0.6289	0.079*	
C14	−0.5079 (11)	1.1370 (5)	0.7412 (4)	0.0549 (16)	
H14	−0.4450	1.0857	0.6938	0.066*	
C15	0.8230 (15)	0.4970 (7)	1.0500 (8)	0.107 (3)	
H15A	0.7983	0.4329	1.0048	0.160*	
H15B	0.8185	0.4763	1.1246	0.160*	
H15C	0.9970	0.5302	1.0405	0.160*	
H2	−0.076 (13)	1.017 (5)	0.7543 (17)	0.080*	
H5A	0.211 (5)	0.997 (5)	0.058 (4)	0.080*	
H5B	0.490 (7)	0.992 (5)	0.054 (4)	0.080*	

### Atomic displacement parameters (Å^2^)

**Table:**

	*U*^11^	*U*^22^	*U*^33^	*U*^12^	*U*^13^	*U*^23^
O1	0.063 (3)	0.055 (2)	0.057 (2)	0.014 (2)	0.001 (2)	0.002 (2)
O2	0.071 (3)	0.059 (2)	0.090 (3)	0.024 (2)	−0.013 (3)	0.007 (2)
O3	0.056 (3)	0.081 (3)	0.032 (2)	0.017 (2)	0.0050 (18)	0.007 (2)
O4	0.090 (4)	0.068 (3)	0.086 (3)	0.044 (3)	−0.002 (3)	−0.005 (2)
O5	0.054 (3)	0.122 (4)	0.038 (2)	−0.011 (3)	0.003 (2)	0.005 (2)
N1	0.042 (3)	0.043 (3)	0.051 (3)	0.004 (2)	−0.004 (2)	−0.003 (2)
N2	0.048 (3)	0.050 (3)	0.039 (3)	0.009 (2)	0.007 (2)	0.002 (2)
C1	0.043 (3)	0.046 (3)	0.057 (4)	0.004 (3)	−0.004 (3)	−0.022 (3)
C2	0.041 (4)	0.046 (3)	0.050 (3)	0.001 (3)	−0.001 (3)	−0.014 (3)
C3	0.047 (4)	0.047 (3)	0.076 (5)	0.010 (3)	−0.012 (4)	−0.010 (3)
C4	0.056 (5)	0.063 (4)	0.097 (6)	0.025 (4)	−0.001 (4)	−0.027 (4)
C5	0.052 (4)	0.076 (5)	0.091 (6)	0.011 (4)	0.011 (4)	−0.035 (4)
C6	0.048 (3)	0.065 (4)	0.064 (4)	0.001 (3)	0.000 (3)	−0.017 (3)
C7	0.050 (4)	0.055 (4)	0.041 (3)	0.003 (3)	0.001 (3)	−0.009 (3)
C8	0.034 (3)	0.042 (3)	0.051 (4)	−0.001 (3)	0.003 (3)	−0.005 (3)
C9	0.041 (3)	0.040 (3)	0.042 (3)	0.003 (3)	0.002 (3)	−0.002 (2)
C10	0.065 (4)	0.068 (4)	0.043 (3)	0.009 (4)	−0.001 (3)	−0.007 (3)
C11	0.078 (5)	0.070 (4)	0.059 (5)	0.037 (4)	0.008 (4)	−0.014 (3)
C12	0.052 (4)	0.049 (3)	0.064 (4)	0.017 (3)	−0.004 (3)	−0.001 (3)
C13	0.076 (5)	0.072 (4)	0.047 (4)	0.023 (4)	−0.003 (3)	0.004 (3)
C14	0.057 (4)	0.063 (4)	0.044 (3)	0.025 (3)	0.001 (3)	−0.007 (3)
C15	0.066 (5)	0.088 (5)	0.160 (8)	0.039 (4)	−0.015 (5)	0.028 (5)

### Geometric parameters (Å, °)

**Table:**

O1—C2	1.352 (6)	C4—H4A	0.93
O1—H1	0.82	C5—C6	1.367 (8)
O2—C3	1.377 (7)	C5—H5	0.93
O2—C15	1.459 (7)	C6—H6	0.93
O3—C8	1.240 (6)	C7—H7	0.93
O4—C12	1.361 (6)	C8—C9	1.480 (7)
O4—H4	0.82	C9—C10	1.378 (7)
O5—H5A	0.86 (3)	C9—C14	1.388 (6)
O5—H5B	0.84 (4)	C10—C11	1.361 (7)
N1—C7	1.279 (6)	C10—H10	0.93
N1—N2	1.385 (6)	C11—C12	1.388 (8)
N2—C8	1.328 (6)	C11—H11	0.93
N2—H2	0.90 (3)	C12—C13	1.369 (7)
C1—C6	1.388 (7)	C13—C14	1.381 (7)
C1—C2	1.407 (7)	C13—H13	0.93
C1—C7	1.440 (7)	C14—H14	0.93
C2—C3	1.407 (7)	C15—H15A	0.96
C3—C4	1.362 (8)	C15—H15B	0.96
C4—C5	1.379 (8)	C15—H15C	0.96
			
C2—O1—H1	109.5	C1—C7—H7	118.5
C3—O2—C15	116.3 (5)	O3—C8—N2	122.1 (5)
C12—O4—H4	109.5	O3—C8—C9	120.7 (5)
H5A—O5—H5B	108 (3)	N2—C8—C9	117.1 (5)
C7—N1—N2	115.5 (4)	C10—C9—C14	117.0 (5)
C8—N2—N1	120.4 (4)	C10—C9—C8	120.5 (5)
C8—N2—H2	121 (4)	C14—C9—C8	122.5 (5)
N1—N2—H2	118 (4)	C11—C10—C9	122.5 (5)
C6—C1—C2	119.2 (5)	C11—C10—H10	118.8
C6—C1—C7	119.0 (6)	C9—C10—H10	118.8
C2—C1—C7	121.8 (5)	C10—C11—C12	119.7 (5)
O1—C2—C3	117.8 (5)	C10—C11—H11	120.2
O1—C2—C1	123.7 (5)	C12—C11—H11	120.2
C3—C2—C1	118.5 (5)	O4—C12—C13	121.6 (5)
C4—C3—O2	126.4 (6)	O4—C12—C11	119.0 (5)
C4—C3—C2	120.7 (6)	C13—C12—C11	119.4 (5)
O2—C3—C2	112.9 (5)	C12—C13—C14	120.0 (6)
C3—C4—C5	120.5 (6)	C12—C13—H13	120.0
C3—C4—H4A	119.7	C14—C13—H13	120.0
C5—C4—H4A	119.7	C13—C14—C9	121.5 (5)
C6—C5—C4	120.0 (6)	C13—C14—H14	119.3
C6—C5—H5	120.0	C9—C14—H14	119.3
C4—C5—H5	120.0	O2—C15—H15A	109.5
C5—C6—C1	121.1 (6)	O2—C15—H15B	109.5
C5—C6—H6	119.4	H15A—C15—H15B	109.5
C1—C6—H6	119.4	O2—C15—H15C	109.5
N1—C7—C1	122.9 (5)	H15A—C15—H15C	109.5
N1—C7—H7	118.5	H15B—C15—H15C	109.5

### Hydrogen-bond geometry (Å, °)

**Table:**

*D*—H···*A*	*D*—H	H···*A*	*D*···*A*	*D*—H···*A*
O1—H1···N1	0.82	1.99	2.687 (5)	142
N2—H2···O5^i^	0.90 (3)	1.99 (3)	2.820 (5)	154 (5)
O4—H4···O2^ii^	0.82	2.15	2.872 (7)	147
O5—H5A···O3^iii^	0.86 (3)	1.97 (3)	2.769 (6)	156 (6)
O5—H5B···O3^iv^	0.84 (4)	2.01 (3)	2.769 (6)	148 (5)
C7—H7···O5^i^	0.93	2.48	3.229 (7)	138
C14—H14···O5^i^	0.93	2.34	3.218 (7)	158

Symmetry codes: (i) *x*−1/2, −*y*+2, *z*+1/2; (ii) *x*−3/2, −*y*+2, *z*−1/2; (iii) *x*, *y*, *z*−1; (iv) *x*+1, *y*, *z*−1.
